# Effects of Serum Potassium on Mortality in Patients With ST-Elevation Myocardial Infarction

**DOI:** 10.7759/cureus.61126

**Published:** 2024-05-26

**Authors:** Colton J Jensen, Jonathan K Nielsen, Matthew M Talbott, Danielle O'Connell, Vivek S Patel, Peyton A Armstrong, Zubaid Rafique, Lillian M Tia, Krishna K Paul, Dietrich V Jehle

**Affiliations:** 1 Department of Emergency Medicine, University of Texas Medical Branch at Galveston, Galveston, USA; 2 Department of Emergency Medicine, Baylor College of Medicine, Houston, USA

**Keywords:** ventricular dysrhythmia, st-elevation myocardial infarction, emergency medicine, cardiology, potassium

## Abstract

Introduction: Disturbances in potassium levels can induce ventricular arrhythmias and heighten mortality in patients with ST-elevation myocardial infarction (STEMI). This study evaluates the influence of sK levels on seven-day mortality and incidence of ventricular arrhythmias in STEMI patients to further improve clinical guidelines and outcomes.

Methods: This retrospective, propensity-matched study analyzed approximately 250,000 acute STEMI patients from 55 major academic medical centers/healthcare organizations (HCOs) in the US Collaborative Network of the TriNetX database. The sK levels recorded on the day of STEMI diagnosis were categorized into four cohorts: sK ≤ 3.4 (hypokalemia), 3.5 ≤ sK ≤ 4.5 (normal-control), 4.6 ≤ sK ≤ 5.0 (high-normal), and sK ≥ 5.1 (hyperkalemia). Patient cohorts were propensity-matched using linear and logistic regression for demographics. Outcomes of seven-day mortality, ventricular tachycardia (VT), and ventricular fibrillation (VF) were compared between these cohorts and the control group.

Results: The analysis showed hypokalemia was linked to significantly higher seven-day mortality (7.2% vs. 4.3%; RR 1.69; p<0.001), and increased rates of VT and VF. Similarly, hyperkalemia was associated with elevated mortality (12.7% vs. 4.6%; RR 2.76; p<0.001), VT, and VF rates. High-normal sK levels showed increased mortality (7.4% vs. 4.7%; RR 1.58; p<0.001), but unchanged VT or VF rates compared to the normal sK group.

Conclusion: This comprehensive study highlights the correlation of sK levels with death in STEMI patients, revealing a nearly doubled risk of mortality with hypokalemia and almost triples with hyperkalemia. More notably, the mortality for STEMIs is higher for high-normal vs normal sK values. Additionally, hypokalemia and hyperkalemia were found to significantly elevate VT and VF risks.

## Introduction

In the United States, approximately 605,000 individuals experience new myocardial infarctions (MI) each year, while 200,000 people suffer from recurrent MI with the overall prevalence of MI being 3.1% in individuals ≥20 years of age [1]. ST-elevation myocardial infarctions (STEMI) make up slightly over half of acute myocardial infarctions (AMI) [2]. In the setting of STEMI, the 30-day mortality rate ranges between 2.5% to 10% due to a multitude of mechanical and electrical complications [3,4]. Disturbances in serum potassium (sK) homeostasis have been linked to increased mortality due to dysrhythmias after AMI [5-9] as cardiac muscle and other electrolyte-sensitive tissue depend on its homeostasis for optimal function [10].

ACC/AHA guidelines recommended maintenance of sK levels above 4.0 mEq/L and sometimes in the range of 4.5-5.0 mEq/L in patients suffering from AMI, as levels below 3.5 mEq/L were found to increase the risk of ventricular dysrhythmias [11-13]. However, with the advent of beta blockers and rapid invasive reperfusion measures, adverse events captured in previous studies have decreased significantly [14-16]. Nevertheless, studies conducted within the last decade provide conflicting data concerning optimal serum potassium in AMI [12,15,17-19]. Accordingly, recent ACS guidelines from the American College of Cardiology and the American Heart Association omit definitive clinical guidelines for serum potassium levels [20,21].

Although recent meta-analyses, such as those performed by Xi et al. [16] and Colombo et al. [14] have attempted to reconcile inconsistencies and compensate for underpowered trials, uncertainty remains concerning optimal sK in the setting of STEMI. Given the conflicting data, this study aims to evaluate the effect of different ranges of sK on ventricular arrhythmias and seven-day mortality to establish an association between ideal sK ranges and optimal outcomes in patients with an acute STEMI.

Abstract presented virtually at the Texas College of Emergency Physicians (TCEP) Research Forum on April 17, 2023.

## Materials and methods

The TriNetX database was selected for this study due to its robust international health research network, which grants access to retrospective electronic medical records encompassing comprehensive diagnostic, procedural, medication, laboratory, and genomic information. The United States Collaborative Network database was utilized which includes nearly 91 million patients across 55 healthcare organizations, primarily consisting of large academic tertiary care centers in America, along with their associated satellite facilities.

Patients of any age were included if they had a visit between December 13, 2002, and December 13, 2022, with any of the ICD codes containing STEMI (11 codes - ICD10:I21. xx) and had a sK value available on the day of diagnosis; see supplementary appendix. Four cohorts of STEMI patients were identified based on sK levels. Cohorts 1-3 were identified as hypokalemia (sK ≤ 3.4 mEq/L), high normal (sK 4.6-5.0 mEq/L), and hyperkalemia (sK ≥ 5.1 mEq/L), respectively. Cohort 4 was identified as low normal (sK 3.5-4.5 mEq/L), and this represents the control group. These rounded cut-offs were chosen for ease of interpretation. Patients were excluded if they had missing potassium values, or if the measured outcomes occurred prior to STEMI diagnosis. TriNetX stores data within 20 years from the time of analysis, so all events prior to December 13, 2002, were excluded.

The primary outcome was 7-d mortality, while the secondary outcomes were rates of ventricular tachycardia (VT - ICD10:I47.2), and rates of ventricular fibrillation (VF - ICD10:I49.01) within seven days of presentation. Seven-day mortality represents any death that occurred from day 0 (same day as diagnosis of STEMI) to seven days later. Outcomes were compared between the low normal (control) cohort and the other cohorts using the TriNetX research platform. The incidence, risk ratios (RR), 95% confidence intervals (CI), and probability values (p) were calculated for the outcomes of interest using the Measure of Association Tool. Cohorts were matched based on age, sex, race, and ethnicity. The option within TriNetX was selected to exclude patients who had outcomes that occurred before the time frame studied. Mortality data within the TriNetX platform is obtained from EMR data and HCOs, in conjunction with the national death registries. There is potential for missed death events when a patient is treated at an HCO not affiliated with the TriNetX network and subsequently experiences a fatal outcome outside of this network. However, this represents only a minor issue, as currently, 94% of Health Care Organizations (HCOs) within the TriNetX network are also linked to the United States death registries. This percentage is steadily increasing as more HCOs continue to be linked with the registries.

The chi-square test and T-test were used for univariate analysis. A propensity score of 1:1 matching was performed on demographics. Propensity scores were generated using greedy nearest neighbor algorithms utilizing a caliper width of 0.1 pooled standard deviations. Balance on covariates was assessed using standardized mean difference, and absolute values >0.1 were considered positive for residual imbalance. TriNetX platform utilizes input matrices of user-identified covariates and conducts linear and logistic regression analysis to obtain propensity scores for individual subjects. TriNetX randomizes the order of rows to eliminate bias resulting from nearest-neighbor algorithms. This study methodology has been previously validated [22,23]. A two-sided alpha of <0.05 was defined as statistical significance. Utilization of the data from TriNetX does not require UTMB IRB review as this is a secondary analysis of de-identified data. The UTMB IRB determined that this project is considered “not human subjects research.”

## Results

A total of 252,652 patients with a diagnosis of STEMI were identified after exclusions with a mean age of 71 (±14 years), of which 37% were female, 12% African American, 72% Caucasian, 71% Hispanic, and 2% Asian. The most common comorbidities were essential hypertension (79%), hyperlipidemia (65%), and diabetes mellitus (42%). Seven-day mortality and ventricular arrhythmia rates were 5.93% and 4.35%, respectively. The median sK was 4.1 mEq/L (IQR).

Before propensity matching, the hypokalemia, low normal, high normal, and hyperkalemia cohorts consisted of 54,868, 214,674, 64,068, and 32,544 patients, respectively. Several STEMI patients had multiple potassium level measurements taken on their day of diagnosis, potentially categorizing them into several potassium groups. After propensity matching, there were 53,401 patients in each cohort for the comparison of the hypokalemia and low normal cohorts. The comparison of low normal with high normal resulted in 61,096 patients in each group, while comparisons of low normal and hyperkalemia generated 30,831 patients in each cohort after propensity matching. Pre- and post-propensity matching demographic values can be found in Tables 1, 2.

**Table 1:** Demographics before propensity score matching - comparisons of experimental cohorts 1-3 vs control cohort 4 low normal potassium (sK 3.5-4.5)

Cohort		Hypokalemia (N=53,401) cohort 1		High normal potassium (N=61,096) cohort 2		Hyperkalemia (N=30,831) cohort 3	
		Patients (%)	p-value	Patients (%)	p-value	Patients (%)	p-value
1-3	Age at index	53,401 (100%)	<0.001	61,096 (100%)	<0.001	30,831 (100%)	<0.001
4_(Control)_		205,648 (100%)		205,648 (100%)		205,648 (100%)	
1-3	Male	28,939 (54.2%)	<0.001	39,380 (64.5%)	<0.001	19,718 (64.0%)	<0.001
4		126,110 (61.3%)		126,110 (61.3%)		126,110 (61.3%)	
1-3	Female	24,148 (45.2%)	<0.001	21,084 (34.5%)	<0.001	10,851 (35.2%)	<0.001
4		77,286 (37.6%)		77,286 (37.6%)		77,286 (37.6%)	
1-3	Not Hispanic or Latino	44,065 (82.5%)	<0.001	49,716 (81.4%)	0.002	24,688 (80.1%)	0.003
4		166,165 (80.8%)		166,165 (80.8%)		166,165 (80.8%)	
1-3	White	38,628 (72.3%)	<0.001	45,096 (73.8%)	0.007	21,516 (69.8%)	<0.001
4		152,917 (74.4%)		152,917 (74.4%)		152,917 (74.4%)	
1-3	Black or African American	9,043 (16.9%)	<0.001	9,364 (15.3%)	0.017	5,636 (18.3%)	<0.001
4		30,714 (14.9%)		30,714 (14.9%)		30,714 (14.9%)	
1-3	Unknown Ethnicity	7,100 (13.3%)	<0.001	8,788 (14.4%)	0.18	4,588 (14.9%)	0.195
4		0,028 (14.6%)		30,028 (14.6%)		30,028 (14.6%)	
1-3	Unknown race	4,641 (8.7%)	0.490	5,416 (8.9%)	0.039	3,034 (9.8%)	<0.001
4		7,679 (8.6%)		17,679 (8.6%)		17,679 (8.6%)	
1-3	Hispanic or Latino	2,236 (4.2%)	<0.001	2,592 (4.2%)	<0.001	1,555 (5.0%)	0.001
4		9,455 (4.6%)		9,455 (4.6%)		9,455 (4.6%)	
1-3	Asian	798 (1.5%)	0.079	907 (1.5%)	0.043	470 (1.5%)	0.318
4		3,292 (1.6%)		3,292 (1.6%)		3,292 (1.6%)	
1-3	American Indian or Alaska Native	254 (0.5%)	0.163	271 (0.4%)	0.674	149 (0.5%)	0.193
4		886 (0.4%)		886 (0.4%)		886 (0.4%)	
1-3	Native Hawaiian or Other Pacific Islander	37 (0.1%)	0.525	42 (0.1%)	0.475	26 (0.1%)	0.703
4		160 (0.1%)		160 (0.1%)		160 (0.1%)	

**Table 2:** Demographics after propensity score matching - comparisons of experimental cohorts 1-3 vs control cohort 4 low normal potassium (sK 3.5-4.5)

Cohort		Hypokalemia (N=53,401) cohort 1		High normal potassium (N=61,096) cohort 2		Hyperkalemia (N=30,831) cohort 3	
		Patients (%)	p-value	Patients (%)	p-value	Patients (%)	p-value
1-3	Age at index	53,401 (100%)	0.947	61,096 (100%)	0.983	30,831 (100%)	0.919
4_(Control)_		53,401 (100%)		61,096 (100%)		30,831 (100%)	
1-3	Male	28,939 (54.2%)	0.985	39,380 (64.5%)	0.943	19,718 (64.0%)	0.953
4		28,936 (54.2%)		39,392 (64.5%)		19,725 (64.0%)	
1-3	Female	24,148 (45.2%)	0.975	21,084 (34.5%)	0.976	10,851 (35.2%)	0.966
4		24,153 (45.2%)		21,079 (34.5%)		10,846 (35.2%)	
1-3	Not Hispanic or Latino	44,065 (82.5%)	1.000	49,716 (81.4%)	0.936	24,688 (80.1%)	0.96
4		44,065 (82.5%)		49,727 (81.4%)		24,693 (80.1%)	
1-3	White	38,628 (72.3%)	0.945	45,096 (73.8%)	0.943	21,516 (69.8%)	0.951
4		38,638 (72.4%)		45,107 (73.8%)		21,523 (69.8%)	
1-3	Black or African American	9,043 (16.9%)	0.948	9,364 (15.3%)	0.981	5,636 (18.3%)	0.975
4		9,035 (16.9%)		9,361 (15.3%)		5,639 (18.3%)	
1-3	Unknown Ethnicity	7,100 (13.3%)	0.986	8,788 (14.4%)	0.993	4,588 (14.9%)	0.964
4		7,098 (13.3%)		8,787 (14.4%)		4,592 (14.9%)	
1-3	Unknown race	4,641 (8.7%)	0.862	5,416 (8.9%)	0.928	3,034 (9.8%)	0.989
4		4,657 (8.7%)		5,425 (8.9%)		3,033 (9.8%)	
1-3	Hispanic or Latino	2,236 (4.2%)	0.976	2,592 (4.2%)	0.887	1,555 (5.0%)	0.868
4		2,238 (4.2%)		2,582 (4.2%)		1,546 (5.0%)	
1-3	Asian	798 (1.5%)	0.820	907 (1.5%)	0.906	470 (1.5%)	0.844
4		789 (1.5%)		912 (1.5%)		476 (1.5%)	
1-3	American Indian or Alaska Native	254 (0.5%)	0.823	271 (0.4%)	0.571	149 (0.5%)	0.77
4		249 (0.5%)		258 (0.4%)		144 (0.5%)	
1-3	Native Hawaiian or Other Pacific Islander	37 (0.1%)	0.632	42 (0.1%)	0.299	26 (0.1%)	0.123
4		33 (0.1%)		33 (0.1%)		16 (0.1%)	

The primary outcome of 7-d mortality before propensity matching was lowest in the low normal potassium cohort (5.128%) while they were 9.073%, 8.376%, and 14.798% for hypokalemia, high normal potassium, and hyperkalemia cohorts, respectively. When compared to low normal potassium, the RR of hypokalemia, high normal potassium, and hyperkalemia cohorts were 1.769 (p<0.001), 1.633 (p<0.001), and 2.866 (p<0.001), respectively. After propensity matching, the patients in the hypokalemia cohort (K ≤ 3.4 mEq/L) had a 7.2% mortality rate with seven days of STEMI, which was significantly higher than those in the low normal range (3.5-4.5 mEq/L) control group, which was at 4.3% (RR 1.69; p<0.001). At that level, hypokalemia was also associated with significantly higher rates of VT (7.5% vs. 5.8%; RR 1.30; p<0.001) and VF (5.9% vs. 3.1%; RR 1.92; p<0.001) when compared to the control group. Similarly, hyperkalemia (K ≥ 5.1 mEq/L) was associated with higher seven-day mortality (12.7% vs. 4.6%; RR 2.76; p<0.001), VT (7.1% vs. 6.1%; RR 1.16; p<0.001), and VF (4.0% vs. 3.1%; RR 1.29; p<0.001) compared to the control group.

**Figure 1:**
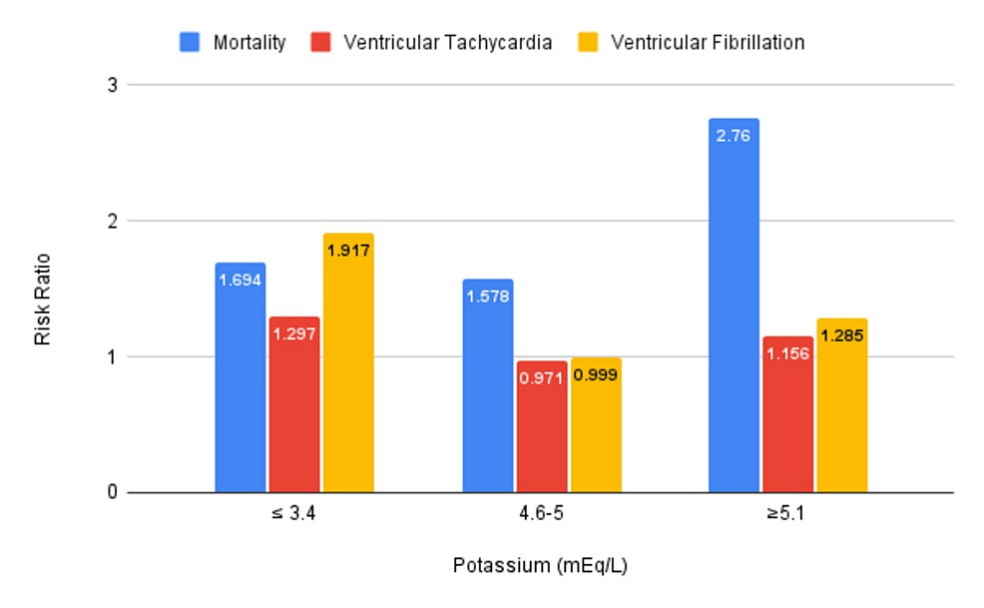
Outcomes for experimental potassium vs. low normal potassium (3.5-4.5) after propensity score matching

Additionally, STEMI patients with high normal sK (4.6 mEq/L ≤ K ≤ 5.0 mEq/L) were associated with a significant increase in seven-day mortality of 7.4% compared to 4.7% in the low normal control group (RR 1.58; p<0.001). However, this range of potassium had no statistically significant increase in VT episodes (6.0% vs. 6.2%; RR 0.971; p=0.203) or VF episodes (3.1% vs. 3.1%; RR 1.00; p=0.967) when compared to the low normal potassium range. All the mortality rates, VF rates, and VT rates pre- and post-propensity matching, along with RRs with 95% CIs and p-values are presented in Tables 3, 4, and Figure 1.

**Table 3:** Potassium levels and outcomes of STEMI patients before propensity score matching VT: ventricular tachycardia; VF: ventricular fibrillation; CI: confidence interval; STEMI: ST-elevation myocardial infarction

Potassium (mEq/L)	Mortality (%)	Risk ratio (95% CI)	Probability (p)
≤3.4	9.1%	1.769 (1.709, 1.832)	<0.0001
3.5-4.5	5.1%		
4.6-5	8.4%	1.633 (1.580, 1.688)	<0.0001
3.5-4.5	5.1%		
≥5.1	14.8%	2.886 (2.791, 2.985)	<0.0001
3.5-4.5	5.1%		
Potassium (mEq/L)	VT (%)		
≤3.4	9.0%	1.337 (1.292, 1.384)	<0.0001
3.5-4.5	6.7%		
4.6-5	6.6%	0.973 (0.939, 1.009)	0.142
3.5-4.5	6.7%		
≥5.1	7.7%	1.140 (1.090, 1.193)	<0.0001
3.5-4.5	6.7%		
Potassium (mEq/L)	VF (%)		
≤3.4	7.8%	2.144 (2.061, 2.229)	<0.0001
3.5-4.5	3.6%		
4.6-5	3.6%	0.994 (0.947, 1.043)	0.795
3.5-4.5	3.6%		
≥5.1	4.6%	1.263 (1.192, 1.338)	<0.0001
3.5-4.5	3.6%		

**Table 4:** Potassium levels and outcomes of STEMI patients after propensity score matching VT: ventricular tachycardia; VF: ventricular fibrillation; CI: confidence interval; STEMI: ST-elevation myocardial infarction

Potassium (mEq/L)	Mortality (%)	Risk ratio (95% CI)	Probability (p)
≤3.4	7.2%	1.694 (1.610, 1.782)	<0.001
3.5-4.5	4.3%		
4.6-5	7.4%	1.578 (1.507, 1.652)	<0.001
3.5-4.5	4.7%		
≥5.1	12.7%	2.760 (2.602, 2.928)	<0.001
3.5-4.5	4.6%		
Potassium (mEq/L)	VT (%)		
≤3.4	7.5%	1.297 (1.238, 1.358)	<0.001
3.5-4.5	5.8%		
4.6-5	6.02%	0.971 (0.927, 1.016)	0.203
3.5-4.5	6.2%		
≥5.1	7.1%	1.156 (1.086, 1.230)	<0.001
3.5-4.5	6.1%		
Potassium (mEq/L)	VF (%)		
≤3.4	5.9%	1.917 (1.807, 2.034)	<0.001
3.5-4.5	3.1%		
4.6-5	3.1%	0.999 (0.938, 1.064)	0.967
3.5-4.5	3.1%		
≥5.1	4.0%	1.285 (1.182, 1.397)	<0.001
3.5-4.5	3.1%		

## Discussion

This study represents the largest investigation to date examining the outcomes associated with potassium derangements in patients with STEMI and this enhances the generalizability of our results. Our findings revealed that the low normal cohort (3.5 mEq/L ≤ K ≤ 4.5 mEq/L) had the lowest 7-d mortality. Notably, the mortality increased even when sK was in the high normal range (4.6 mEq/L ≤ K ≤ 5.0 mEq/L) with an RR of 1.58 when compared to the low normal cohort. Last, the mortality was highest in the hyperkalemia cohort with close to three times the risk of death compared to the low normal cohort. As for ventricular arrhythmia, the rates were statistically significant only in the hypokalemia and hyperkalemia cohorts when compared to the low normal cohort.

Increased mortality associated with hyperkalemia has been described in multiple studies [24-27], which is consistent with our findings. Einhorn et al. reported the first study associating the incidence of hyperkalemia with increased mortality within 24 hours of hospitalization. However, increased mortality and adverse outcomes in cardiac patients with sK abnormalities, particularly those in the high normal range, have been demonstrated in only a few studies [15,28,29]. Goyal et al. showed that sK > 4.5 led to twice the mortality rate (OR 1.99) and even greater for higher sK levels in AMI patients - consistent with results from our study [15]. Additionally, Zhang et al. showed mortality is highest when the mean potassium level is ≥5.5 (OR 15.8) [28]. Discrepancies in OR values might be attributed to differences in sK category cutoffs and the measurement of in-hospital mortality compared to overall 7-d mortality.

Increased rates of ventricular dysrhythmia have likewise been described [15,19]. Goyal et al. and Xi et al. demonstrated that rates of arrhythmia were highest among AMI patients with overt hypokalemia or hyperkalemia - creating a U/J-shaped curve. Our data support these findings, indicating that extremes of sK levels correlate with increased mortality and the incidence of life-threatening ventricular dysrhythmias in the context of STEMI. Patel et al., similarly, described increased rates of ventricular arrhythmia at extremes of the potassium range but also reported increases within high normal sK ranges [19]. This difference might be explained by their addition of bradyarrhythmia as an outcome.

This is the first investigation of such magnitude, six times larger than any previous studies, to provide evidence for the relationship of sK in STEMI patients and the use of robust statistical methods such as propensity matching reinforces the reliability of these results. Of particular interest are the STEMI patients in the high normal range (4.6 mEq/L ≤ K ≤ 5.0 mEq/L), who have a greater mortality. Although various explanations exist for the correlation between potassium imbalance and mortality including the size of infarction, arrhythmia, heart block, asystole, or renal etiologies [30], our findings suggest that strict control of potassium level might decrease mortality in patients with STEMI - one of the most frequent and deadly medical emergencies of the modern world.

This study has several limitations that should be considered. Due to the retrospective nature of our study, we were able to demonstrate an association between potassium levels and outcomes, but not causation. One limitation was the limited number of arbitrary independent variable cohorts. The absence of multiple discrete groups across the entire span of the sK range limits our ability to observe differences across extremes of the potassium spectrum. For example, in future studies differentiation of severe vs moderate hyperkalemia should be included by organizing a cohort specifically for severe potassium levels ≥7.5 mEq/L, to delineate between outcomes for moderately and severely elevated levels. Another limitation is the absence of data on sK management during hospitalization. Additionally, not all VT/VF events may have been recorded due to various reasons, potentially leading to an underestimation of the true incidence. The correlation of high normal potassium levels with increased mortality in STEMI patients does not necessarily imply that correcting the potassium levels decreases death rates. This association may only reflect greater potassium leakage in larger infarctions and outcomes may not necessarily be improved by reducing the potassium levels. Last, because of the observational nature of this study and despite all the statistical adjustments, there is a possibility of residual confounding by underlying conditions such as renal failure which may lead to higher sK levels that could contribute to mortality.

## Conclusions

In this large study, we set out to investigate the relationship between hypokalemia and hyperkalemia with mortality and ventricular arrhythmia in patients with STEMI. Our findings revealed that mortality nearly doubles with hypokalemia and almost triples with hyperkalemia. The rise in mortality at sK >4.5 raises concerns regarding the ideal potassium range when caring for patients with STEMI. The results of this study have the potential to help develop future standardized guidelines and identify specific ranges of potassium levels for optimal treatment after STEMI. Prospective studies are indicated to further elucidate this relationship.

## Appendices

**Table 5:** Codes for STEMI and potassium labs ICD-10: International Statistical Classification of Diseases and Related Health Problems - 10th Revisions; TNX Curated: TriNetX Curated - Composite code created by TriNetX; STEMI: ST-elevation myocardial infarction

Name	Codes
ST elevation (STEMI) myocardial infarction of the anterior wall	ICD-10: I21.0
ST elevation (STEMI) myocardial infarction involving the left main coronary artery	ICD-10: I21.01
ST elevation (STEMI) myocardial infarction involving left anterior descending coronary artery	ICD-10: I21.02
ST elevation (STEMI) myocardial infarction involving another coronary artery of the anterior wall	ICD-10: I21.09
ST elevation (STEMI) myocardial infarction of interior wall	ICD-10: I21.1
ST elevation (STEMI) myocardial infarction involving the right coronary artery	ICD-10: I21.11
ST elevation (STEMI) myocardial infarction involving another coronary artery of the interior wall	ICD-10: I21.19
ST elevation (STEMI) myocardial infarction of other sites	ICD-10: I21.2
ST elevation (STEMI) myocardial infarction involving left circumflex coronary artery	ICD-10: I21.21
ST elevation (STEMI) myocardial infarction involving other sites	ICD-10: I21.29
ST elevation (STEMI) myocardial infarction of unspecified site	ICD-10: I21.3
Potassium (moles/volume) in serum, plasma, or blood	TNX Curated: 9028

Greedy nearest-neighbor matching

The most common implementation of propensity matching is pair-matching, in which pairs of treated and control participants are formed. There are several common implementations of pair matching. The most commonly used is greedy nearest neighbor matching (NNM), which we used, in which a treated participant is selected at random and then matched to the control participant whose propensity score is closest to that of the treated participant. The process is described as greedy because, at each stage, the control is selected based on who is closest to the currently considered treated participant, even if that untreated participant would serve as a better control for a subsequently treated participant. This process is then repeated until a matched control participant has been selected for each treated participant. This process generally uses matching without replacement, so that once a control participant is matched to a treated participant, that control participant is no longer available for matching to a subsequent treated participant. A refinement of NNM is NNM with a caliper restriction. Using this approach, a control participant is an acceptable match for a treated participant only if the difference in their propensity scores is less than a maximum amount (the caliper width or distance). For technical reasons, one typically matches the logit of the propensity score and uses a caliper width that is deﬁned as a proportion of the (0.1-0.2) SD of the logit of the propensity score. A crucial step in any study that uses propensity score matching is to assess the degree to which matching the propensity score resulted in the formation of a matched sample in which the distribution of baseline characteristics is similar between treated and control participants. This assessment is critical as it allows both the researcher and readers to assess whether matching the estimated propensity score has removed systematic baseline differences between treatments. The use of the standardized difference, which is the difference in means in units of SD, is often used for assessing the similarity of matched treated and control participants. Some authors have suggested that a threshold of 0.10 (or 10%) be used to denote an acceptable balance after matching. Once an acceptable balance has been achieved, analysts can unblind themselves to the outcome and compare outcomes between treated and control participants in the matched sample. The analyses conducted in the propensity score-matched sample can be similar to those that would be done in a randomized controlled trial with a similar outcome.
